# Hypothalamic A11 Nuclei Regulate the Circadian Rhythm of Spinal Mechanonociception through Dopamine Receptors and Clock Gene Expression

**DOI:** 10.3390/life12091411

**Published:** 2022-09-10

**Authors:** Celia Piña-Leyva, Manuel Lara-Lozano, Marina Rodríguez-Sánchez, Guadalupe C. Vidal-Cantú, Ericka Barrientos Zavalza, Ismael Jiménez-Estrada, Rodolfo Delgado-Lezama, Leonardo Rodríguez-Sosa, Vinicio Granados-Soto, Juan Antonio González-Barrios, Benjamín Florán-Garduño

**Affiliations:** 1 · Department of Physiology, Biophysics, and Neurosciences, CINVESTAV, Av. No. 2508 National Polytechnic Institute, Mexico City 06760, Mexico; 2 Genomic Medicine Laboratory, Regional Hospital “October 1st”, ISSSTE, Av. No. 1669 National Polytechnic Institute, Mexico City 07760, Mexico; 3 Neurobiology of Pain Laboratory, Departamento de Farmacología, Cinvestav, Sede Sur, México City 14330, Mexico; 4 Doctorado en Ciencias Biológicas y de la Salud, Universidad Autónoma Metropolitana, Unidad Iztapalapa, Mexico City 09340, Mexico; 5 Department of Physiology, Medicine Faculty, National Autonomous University of Mexico, University City, Mexico City 04510, Mexico

**Keywords:** dopaminergic A11 nucleus, paw withdrawal threshold, lumbar spinal cord, clock gene, circadian rhythms, mechanonociception, D1 receptor, D2 receptor

## Abstract

Several types of sensory perception have circadian rhythms. The spinal cord can be considered a center for controlling circadian rhythms by changing clock gene expression. However, to date, it is not known if mechanonociception itself has a circadian rhythm. The hypothalamic A11 area represents the primary source of dopamine (DA) in the spinal cord and has been found to be involved in clock gene expression and circadian rhythmicity. Here, we investigate if the paw withdrawal threshold (PWT) has a circadian rhythm, as well as the role of the dopaminergic A11 nucleus, DA, and DA receptors (DR) in the PWT circadian rhythm and if they modify clock gene expression in the lumbar spinal cord. Naïve rats showed a circadian rhythm of the PWT of almost 24 h, beginning during the night–day interphase and peaking at 14.63 h. Similarly, DA and DOPAC’s spinal contents increased at dusk and reached their maximum contents at noon. The injection of 6-hydroxydopamine (6-OHDA) into the A11 nucleus completely abolished the circadian rhythm of the PWT, reduced DA tissue content in the lumbar spinal cord, and induced tactile allodynia. Likewise, the repeated intrathecal administration of D1-like and D2-like DA receptor antagonists blunted the circadian rhythm of PWT. 6-OHDA reduced the expression of *Clock* and *Per1* and increased *Per2* gene expression during the day. In contrast, 6-OHDA diminished *Clock*, *Bmal*, *Per1*, *Per2*, *Per3*, *Cry1*, and *Cry2* at night. The repeated intrathecal administration of the D1-like antagonist (SCH-23390) reduced clock genes throughout the day (*Clock* and *Per2*) and throughout the night (*Clock*, *Per2* and *Cry1*)*,* whereas it increased *Bmal* and *Per1* throughout the day. In contrast, the intrathecal injection of the D2 receptor antagonists (L-741,626) increased the clock genes *Bmal*, *Per2*, and *Per3* and decreased *Per1* throughout the day. This study provides evidence that the circadian rhythm of the PWT results from the descending dopaminergic modulation of spinal clock genes induced by the differential activation of spinal DR.

## 1. Introduction

The spinal cord has been considered the center that controls peripheral functions’ rhythmicity. Spinal injury disrupts several circadian rhythms through changes in clock genes’ expression, indicating their role in the circadian process [1,2]. Some forms of sensory perception, such as thermal and chemical, have a circadian rhythm [3,4,5]. Importantly, changes in the expression of clock genes in the lumbar spinal medulla produce mechanical hypersensitivity, relating the clock genes’ activity with sensory function [1]. However, to date, it is not known if mechanonociception itself has circadian rhythmicity.

On the other hand, dopamine (DA) and its receptors (mainly D2-like) regulate clock gene expression in retinal ganglion cells and striatum primary cultures, and, as a consequence, the function is controlled by such genes [6,7,8,9,10]. It is well known that several spinal cord regions receive dopaminergic input from the hypothalamic dopaminergic A11 nucleus [11,12,13,14,15], which appears to be responsible for activating DA receptors (DR) [13,16]. Moreover, the D1- and D2-like receptor families are expressed in the dorsal spinal cord [13,17,18]. Additionally, hypothalamic A11 has been related to the regulation of pain [11,19], measured through tactile hypersensitivity [20,21]. It has been shown that extracellular DA tone and tyrosine hydroxylase expression have circadian rhythms [22,23,24]. Thus, it is possible that in normal conditions, mechanonociception in the spinal cord has circadian rhythmicity and DA from the A11 area modulates such sensitivity through DR and changes in clock gene expression. This study used the evaluation of the paw withdrawal threshold (PWT), using the Von Frey hairs as a tool to study the mechanisms of cutaneous stimulation-induced sensory input [25,26,27]. This work aimed to show circadian rhythm in the PWT in normal rats, the role of the A11 area, DA and its receptors’ modulation of the circadian rhythmicity of the PWT, and possible changes in the expression of lumbar spinal clock gene transcription. We found that circadian rhythmicity in rats’ PWT is disrupted by the 6-OHDA lesion of the A11 area, and by the intrathecal DA D1-like and D2 receptor blockade in non-lesioned rats and modification of clock gene expression. Additionally, the content of diurnal DA and DOPAC increases during dusk. These data indicate the physiological role of DA and the A11 area in regulating mechanical-sensitivity rhythmicity in normal conditions.

## 2. Materials and Methods

### 2.1. Animals

Male Wistar rats (210 to 230 g) were obtained from our breeding facilities (CINVESTAV). The animals were maintained in suitable animal rooms under controlled temperature conditions (22 ± 3 °C) and a light–dark cycle (12 h–12 h, light onset at 8:00 a.m.). Food and water were provided ad libitum. After habituation for 2 weeks, the animals were used for the experimental procedures. For A11 lesions, the animals were anesthetized using a mixture of ketamine and xylazine (50/10 mg/kg). The lesion of the dopaminergic A11 neurons was induced via bilateral injection of 6-OHDA (10 µg solved in 1 µL of 0.02% ascorbic acid), stereotaxically administered in the hypothalamic A11 area at coordinates of AP-3.6, ML + 0.06 VL-7.5, according to the atlas of Paxinos and Watson [28]. Thirty minutes earlier, the rats were injected with imipramine (10 mg/kg, i.p.) to protect the noradrenergic fibers [29]. In the control group for 6-OHDA lesions, the animals received an injection of saline. The animals lesioned in this way were tested for PWT, and another group was used for DA and DOPAC determinations or intrathecal DA agonist administration two weeks after surgery. All experimental procedures were conducted according to the current Mexican legislation (NOM-062-ZOO-1999, SAGARPA), the Guide for the Care and Use of Laboratory Animals (NIH), and the Guidelines on Ethical Standards for Investigation of Experimental Pain in Animals [30]. Our local Ethics Committee approved these experiments (Protocol 0146-15, Coinvested, Mexico City, Mexico). Efforts were made to minimize the number of animals used and their suffering.

### 2.2. Experimental Groups and PWT Measurement

**Figure 1:**
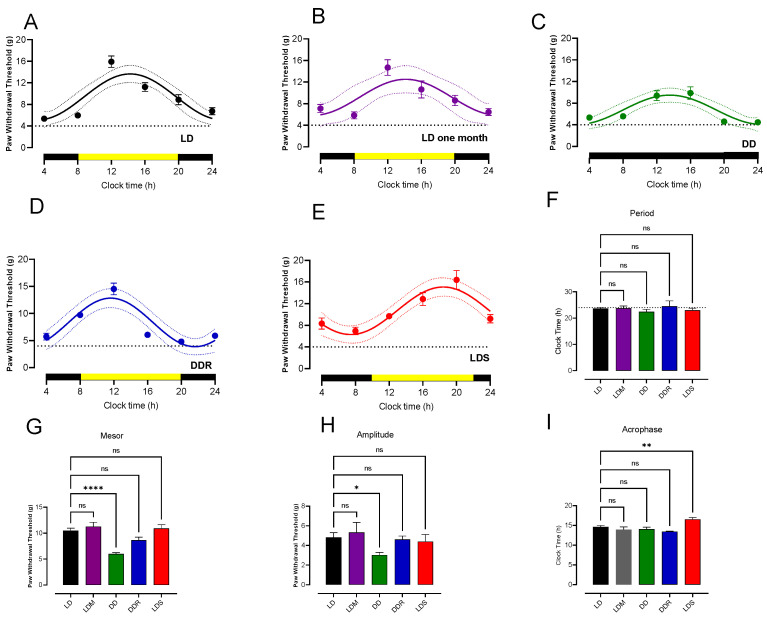
PWT circadian rhythm in male Wistar rats. (**A**) The time course of paw withdrawal threshold (g) in naïve rats in a light–dark cycle (light from 8:00 a.m. to 8:00 p.m. and dark from 8:00 p.m. to 8:00 a.m.) (LD, 12 h–12 h, n = 12). (**B**) Evaluation of PWT in four rats from the LD group one month later (LD one month, 12 h–12 h, n = 4). (**C**) The time course of PWT in naïve rats was maintained in a continuous dark cycle (DD, 24 h, n = 8). (**D**) Evaluation of PWT in four rats from the DD group two weeks after restoring the LD cycle (DDR, 12 h light-12 h dark, n = 4). (**E**) The time course of PWT in rats in which LD cycle was shifted by 2 h (light from 10:00 a.m. to 10:00 p.m. and dark from 10:00 p.m. to 10:00 a.m.) (LDS, 12 h × 12 h, n = 10). Points represent the PWT values in g (mean ± SEM) determined at that time; continuous lines indicate the best sinewave fitted values, and the dotted lines represent the confidence interval. Yellow bars indicate light exposure, and black bars indicate the night. (**F**–**I**) The best-fitted values of the period, mesor, amplitude, and acrophase as mean ± SEM. * *p* < 0.05, ** *p* < 0.01, **** *p* < 0.0001, and ns = no significant differences with respect to LD group. Data were analyzed using one-way ANOVA followed by the Fisher test.

**Figure 2:**
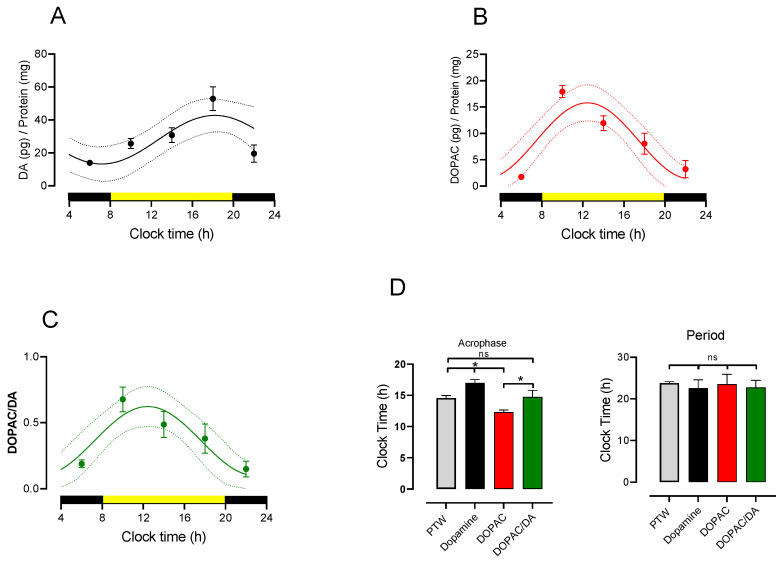
Circadian rhythms of DA, DOPAC, and turnover in the lumbar spinal cord region. (**A**) The temporal course of the lumbar spinal cord region. (**B**) The temporal course of the primary DA metabolite DOPAC in the lumbar region. (**C**) The temporal course of DA turnover in the lumbar spinal cord (expressed as the ratio of the DA/DOPAC contents). Determinations were obtained from four rats at each point. Data are expressed as mean ± SEM. Continuous lines indicate the best sinewave fitted values, and the dotted lines are the confidence intervals. Yellow bars indicate light exposure, and black bars represent the night. (**D**) The mean ± SEM of the best-fitted values of acrophase and period from (**A**–**C**), compared with the values obtained from the temporal course of the PWT control group (Figure 1A). * *p* < 0.05 and ns = no significant differences among indicated comparisons. Data were analyzed using one-way ANOVA followed by the Fisher test.

**Figure 3:**
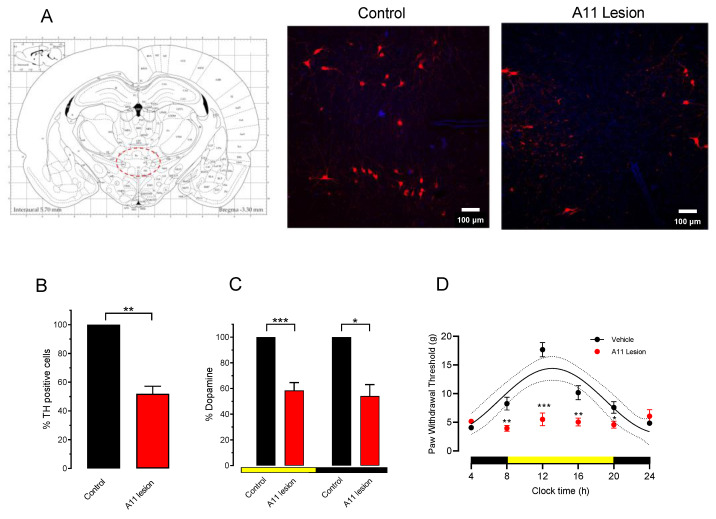
6-OHDA lesion of the A11 area decreases DA content in the lumbar spinal cord region and abolishes PWT rhythms and values. (**A**) Schematic diagram of the location of the A11 area in the midbrain and two representative microphotographs from a control (normal) and a subject with 6-OHDA bilaterally injected into the A11 area. (**B**) TH-positive cells in the A11 area are expressed as the change in percentage of lesioned rats from the control rats (n = 3 rats each group). (**C**) Change in lumbar spinal content of DA, expressed as the change in percentage of lesioned rats from the control rats determined at 10:00 AM and 10:00 PM in a different set of animals (n = 5 rats in each group). * *p* < 0.05, ** *p* < 0.01, *** *p* < 0.001 compared to control in unpaired *t*-test. (**D**) The effect of A11 area lesion on the temporal course of PWT expressed in g (mean ± SEM). Rats were maintained in an LD 12 h–12 h cycle. Black points represent data from 7 rats with the vehicle injected into A11, and red points represent 8 rats injected with 6-OHDA. The continuous line indicates the best sinewave fitted values, and the dotted lines are the confidence intervals. Yellow bars indicate light exposure, and black bars represent the night. * *p* < 0.05, ** *p* < 0.01, *** *p* < 0.001 compared to the vehicle value. Data were analyzed using two-way ANOVA followed by Fisher test.

**Figure 4:**
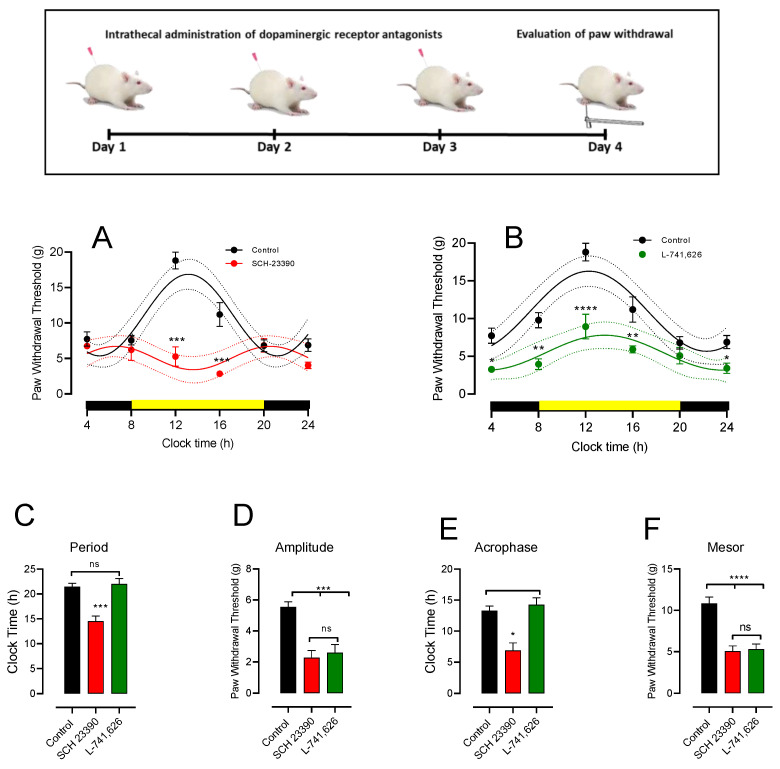
D1-like and D2 receptors regulate the rhythm of PWT and their values. The upper diagram shows the experimental design. (**A**) The effect of D1-like receptor blockade, with SCH23390 (3.24 µg) intrathecally administered in the lumbar spinal cord, on PWT temporal course (n = 5 rats). (**B**) The effect of D2 receptor blockade, with L741,626 (3.40 µg) intrathecally administered in the lumbar spinal cord, on PWT temporal course (n = 5 rats). Black points and curves represent data from rats intrathecally treated with the vehicle (n = 10 rats). The continuous line indicates the best sinewave fitted values, and the dotted lines are the confidence intervals. Yellow and black bars indicate light exposure and night, respectively. * *p* < 0.05, ** *p* < 0.01, *** *p* < 0.001 and **** *p* < 0.0001 with respect to the vehicle value. Data were analyzed using two-way ANOVA followed by Fisher test. (**C**–**F**) represent the best-fitted values of the period, amplitude, acrophase, and mesor for the curves of SCH-23390 and L714,626 from (**A**,**B**), compared with values of the PWT course from rats treated with the vehicle. * *p* < 0.05, ** *p* < 0.001, *** *p* < 0.001, **** *p* < 0.0001 and ns = no significant differences among groups. Data were analyzed using two-way ANOVA followed by Fisher test.

**Figure 5:**
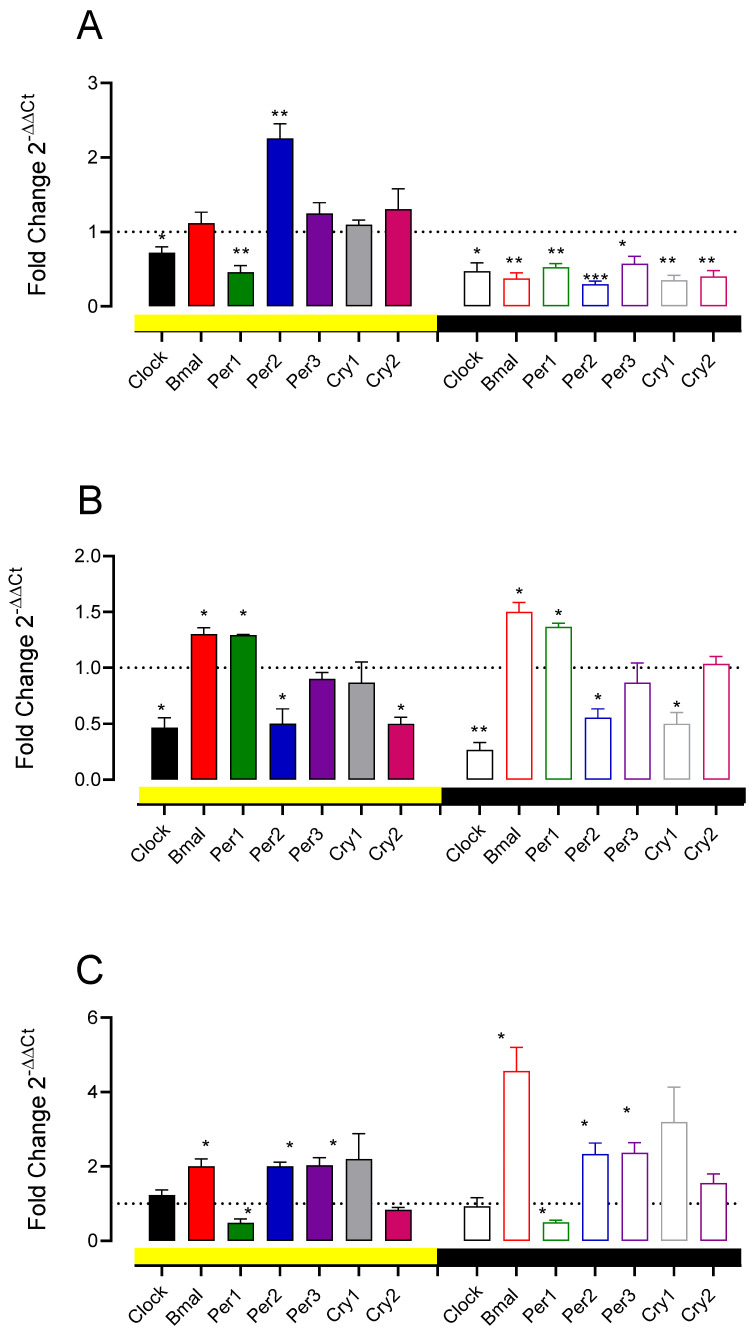
Dopaminergic modulation of expression of clock genes in the lumbar spinal cord. (**A**) The effect of A11 area with 6-OHDA on *Clock*, *Bmal*, *Per1*, *Per2*, *Per3*, *Cry1*, and *Cry2* expression, determined using RTPCR in the lumbar spinal cord, at 10:00 AM (yellow bar) and 10:00 PM (black bar). (**B**,**C**) The effect of intrathecally administered D1-like antagonist SCH23390 and D2 antagonist L741,626 in naïve rats for four days in the same clock genes. Data are expressed as the fold change 2^−ΔΔCt^ mean ± SEM, n = 4 in (**A**–**C**). * *p* < 0.05, ** *p* < 0.01, and *** *p* < 0.001 with respect to theoretical mean 1, one-sample *t*-test.

The paw withdrawal threshold (PWT) was determined as previously described [25]. Briefly, the rats were placed in cages with a mesh grid floor and allowed to acclimate for at least 30 min before experimentation. Subsequently, Von Frey filaments (Stoelting, Wood Dale, IL, USA) were used to determine the threshold of removal of the hind limb (50% paw withdrawal threshold) using the up-and-down method [25,26]. The PWT was evaluated in several groups of rats: (1) a control group (n = 12 rats) maintained in a light–dark (LD) cycle (12 h–12 h, light onset at 8:00 a.m.) (Figure 1A); (2) a control group in which 4 rats returned to their cages and were evaluated one month later (Figure 2A); (3) a dark–dark (DD) group (n = 8) of rats maintained in constant darkness for 15 days to determine whether the PWT could be an intrinsic circadian behavior (the evaluation of the PWT in darkness was performed by using red light, which is invisible to rodents and does not affect the circadian experimental results [31]) (Figure 1C); (4) from the DD group, 4 rats were returned to their cages, placed in the LD condition, and evaluated one month later (Figure 1D); (5) a group (n = 10 rats) in a shifted light–dark (SLD) cycle (12 h–12 h, light onset at 8:00 a.m., Figure 1E); (6) an A11 lesion group (n = 8) with bilateral administration of 6-OHDA into the dopaminergic A11 nucleus (Figure 3D); (7) an SCH-23390 group (n = 5) of normal rats treated with the DR1/DR5 antagonist intrathecally (Figure 4A); (8) an L-741,626 group (n = 5) treated with the DR2 antagonist intrathecally (Figure 4B); (9) a vehicle (n = 10) intrathecally injected group (Figure 4A,B); and (10) an A11 vehicle (n = 7) injected group (Figure 3D). 

The PWT tests started with a filament of 2 g in the middle of a series of 20 filaments with different rigidity or hardness levels, which was changed according to the animal’s response. The 50% withdrawal threshold was determined according to the following formula: 50% threshold (g) = (10^[Xf + k^^δ]^)/10,000, where Xf is the value of the last von Frey filament used (in logarithmic units); k is the correction factor based on the response patterns of a calibration table and the tabulated value based on the design of positive and negative responses; and δ denotes the average differences between stimuli in logarithmic units. The presence of allodynia occurs when the 50% withdrawal threshold of the limb is <4 g [25].

### 2.3. Determination of DA Content in the Lumbar Spinal Cord of Rats

Twenty-four normal rats were sorted into six groups (n = 4 animals per group). Each group was randomly selected for sacrifice by cervical dislocation at 06:00, 10:00, 14:00, 18:00, 22:00, and 2:00 h, an then they were subsequently decapitated, and their spinal cords removed via negative pressure injection of saline applied using a 20 mL syringe through the vertebral central canal at the spinal sacral level (S2–S3) to obtain the lumbar segment. Each segment was homogenized in 0.1 N perchloric acid (200 µL per sample) and centrifuged at 3000 rpm for 3 min. The pellets were washed and resuspended in 1 N NaOH, and the amount of total protein in each sample was determined using the Bradford method and processed according to Quiroz et al. [32]. The supernatant was analyzed via electrochemical detection using a high-pressure liquid chromatography (HPLC) system [33]. The DA and DOPAC contents were quantified in the control and 6-OHDA-treated rats in the lumbar spinal cord. For DA separation, 25 µL of the sample was injected into a C18 column (particle size 2.7 µm, 4.6 mm wide, and 100 mm long; SUPELCO Analytical from Sigma-Aldrich Co. (St. Louis, MO, USA) using a mobile phase (monochloroacetic acid 100, EDTA 0.507, 1-octane sulfonic acid 0.767 (in mM), and acetonitrile 4.5%; pH NaOH adjusted to 3.2), coupled with electrochemical detection, with a cell potential setting of 0.382 mV. The flow rate was set at 0.9 mL/min and the total run time was 10 min. This method measured the concentration of DA and DOPAC in samples from the lumbar. The data are expressed in pg/mg protein and were determined in eight saline-injected and eight 6-OHDA-lesioned rats for the determination of DA and DOPAC contents, to evaluate the effect of the lesion in the lumbar region (Figure 3). 

### 2.4. Determination of TH in the A11 Nucleus

Each animal was anesthetized and perfused with phosphate-buffered saline (PBS) and a 4% paraformaldehyde solution. Then, the brain was collected and stored in 30% sterile phosphate-buffered sucrose for one day. These samples were sliced using a cryostat (30 μm) and collected free-floating in PBS. Tissue sections were blocked for 2 h in 0.1% Tween-PBS containing 1% bovine serum albumin (BSA) and incubated overnight at 4 °C with an anti-tyrosine hydroxylase antibody (1:500, Abcam, Boston, MA, USA). After washing in 0.1% Tween-PBS, the sections were incubated for 2 h with an anti-sheep IgG-conjugated Alexa 599 antibody (1:500, Abcam, Boston, MA, USA). The cells were observed using a Leica microscope (Leica Microsystem, Mannheim, Germany).

### 2.5. Retrotranscription

According to the manufacturer-suggested protocol, total RNA was extracted from the rat lumbosacral spinal cord region using Trizol reagent (Invitrogen Corporation, Carlsbad, CA, USA). RNA was quantified using spectrophotometry at 260 nm and analyzed using 1% agarose gel electrophoresis. cDNA was obtained from 5 μg of total RNA using a SuperScript III reverse transcriptase kit (1 μL, Catalog 18080093, Invitrogen, Carlsbad, CA), Oligo dT 50 μM (1 μL), dNTP mix 10 mM (1 μL), and 13 μL molecular biology-grade water. The retrotranscription conditions were denaturation at 70 °C for 10 min, hybridization at 42 °C for 5 min, the synthesis of cDNA at 55 °C for 50 min, and then, 70 °C for 15 min, and the removal of RNA at 37 °C for 20 min. Finally, RNase H (1 μL, Invitrogen, Carlsbad, CA, USA) was added, and the samples were incubated at 37 °C for 20 min.

### 2.6. Real-Time PCR Assay

cDNA was used to amplify each gene using TaqMan probes (Thermo Fisher Scientific, Waltham, MA, USA). The amplification reactions contained 0.25 μL of the respective TaqMan probes: β-actin (Rn00667869_m1), *Cry1* (Rn01503063_m1), *Cry2* (Rn01485701_m1), *Per1* (Rn01496757_m1), *Per2* (Rn01427704_m1), *Per3* (Rn00709499_m1), *Bmal* (Rn00577590_m1), *Clock* (Rn00573120_m1); 2.5 μL of Master Mix (TaqMan Universal Master Mix II, Life Technologies, Carlsbad, CA, USA); and 2.25 μL of cDNA to a final volume of 5 μL. The qPCR conditions were: 10 min for denaturation at 9 °C, followed by 45 cycles of amplification for 15 s at 95 °C and 1 min at 60 °C. Rat β-actin was used as an internal control and for normalization. The amplification assays were conducted using a 7900HT Fast Real-Time PCR System (Applied Biosystems, Foster City, CA, USA). The 2^−ΔΔCT^ method was used to calculate the relative transcript levels, expressed as the fold change for gene expression [34].

### 2.7. Drugs

6-Hydroxydopamine hydrochloride (6-OHDA), (±)-3-[4-(4-Chlorophenyl)-4-hydroxypiperidinyl]methylindole (L741,626), imipramine hydrochloride, and R(+)-7-chloro-8-hydroxy-3-methyl-1-phenyl-2,3,4,5-tetrahydro-1H-3-benzazepine hydrochloride (SCH-23390), were obtained from Sigma-Aldrich (St. Louis, MO, USA). 6-OHDA was dissolved in 0.02% ascorbic acid. Imipramine, SCH-23390, and L-741,626 were dissolved in saline. A stock solution of L741,626 (680 µg solved in 1 mL of saline) and SCH-23390 (648 µg solved in 1 mL of saline) was used. After dissolving the drugs, they were diluted with normal saline 1000 times before administration. The final dose of L741,626 was 3.4 µg, and of SCH-23390 was 3.24 µg contained in 5 µL, which was intrathecally administered. All drugs were prepared on the day of the experiment. According to the previously reported method, the drugs were intrathecally injected via direct puncture using a 30-gauge needle [35].

### 2.8. Circadian Analysis

The time-course data are expressed as the mean ± standard error. These data were analyzed using the Friedman test, followed by Dunn’s test. The data were then examined via rhythm analysis using the Chronos-Fit software v1.0 [36]. This software includes a linear analysis (data such as the mean, standard deviation, and area under the curve) and rhythm analysis, including a partial Fourier analysis and a stepwise regression technique. While conventional partial Fourier analysis always consists of all harmonics in the fitted model, ‘rhythm analysis’ fits each harmonic separately and checks significance using the F-test. The most significant harmonic is then included in the model if it improves the existing model significantly (in the example below, at level *p* < 0.05). Fitting of the partial Fourier series is accomplished using the following equation:

$$f\left(t\right)=mesor+\sum_{j=1}^{s}\left(amplitude_{i}\times cos\left(t-acrophase_{i}\right)\times \frac{2\pi }{p_{i}}\right)$$

where *mesor* = the midline estimating statistic of rhythm, *i* = the counter for the harmonics, *S* = the number of included harmonics, *amplitude* = the amplitude of the sinewave, *t* = time, *acrophase* = *acrophase* (time of the maximum of the sinewave), and *p* = the entire period length (usually 24–28 h). The fitted curves and corresponding parameters (*mesor*, *amplitudes*, and *acrophases*) are given for each data set [37]. Additionally, we performed data analysis using a sine wave with nonzero-baseline non-linear regression included in GraphPad Prism version 9.4.0, and compared with those parameters obtained with Chronos-Fit, found a close similarity. The graphs show the best-fitted graph and confidence interval obtained using Prism software.

### 2.9. Statistical Analysis

The PWT values, best-fitted circadian rhythms parameters, DA and DOPAC contents, and clock gene fold change are expressed as the mean ± standard error. Statistical comparisons among three groups were analyzed using one-way ANOVA, followed by the Fisher test, whereas an unpaired Student’s *t*-test was performed to compare the two groups. A one-sample *t*-test was performed to compare clock gene expression to the expected value. A *p*-value less than 0.05 was considered significant.

## 3. Results

### 3.1. Circadian Rhythmicity of Paw Withdrawal Threshold

First, we determined the possible circadian rhythm in the PWT in normal rats maintained in constant light–dark cycles (LD, light–dark; 12 h–12 h). The evaluation of the PWT was carried out every 4 h for 24 h (the experiment started at 8:00 AM and finished at the same time the next day). The rhythmic parameters of the best-fitted curve were the mesor, amplitude, acrophase, and period (first-period length of 20–28 h). The results indicated that rats in the LD group (Figure 1A, n = 12 rats) had PWT values of about 5.97 ± 0.34 g at the beginning of the experiment (8:00 h). This value increased to about 15.92 ± 1.06 g at noon h (four hours later), and after that, it decreased to 5.36 ± 0.45 g at 4:00 h in the morning. Further rhythm analysis of the time course of the PWT revealed a period of 23.65 ± 0.36 h, an acrophase at 14.63 ± 0.33 h, an amplitude of 4.81 ± 0.48 g, and a mesor of 10.49 ± 0.45 g. The mean adjusted Sx.y values from each rat were 2.23 ± 0.34 and R^2^ 0.82 ± 0.03. The mean period value obtained was not statistically different from the 24 h theoretical mean (t = 1.057, df = 11, *p* = 0.313, one-sample *t*-test). A sample of four rats was evaluated one more time one month later to see if they had similar values of PWT throughout the day as before. The rats (Figure 1B, n = 4 rats) had PWT values of about 5.84 ± 0.64 g at the beginning of the experiment (8:00 h). This value increased to about 14.69 ± 1.44 g at noon (4 h later), and after that, it decreased to 7.10 ± 0.76 g at 4:00 h in the morning. These values were very similar to the initial group evaluated. Rhythm analysis indicated no significant differences in sine-wave curve parameters between the groups, as seen in Figure 1F,I. 

To determine if circadian PWT is an intrinsic rhythm from the original group, we conditioned a group of rats to a dark–dark (DD) cycle (n = 8, Figure 1C). The DD group of rats exhibited PWT values of about 4.83 ± 0.50 g at 8:00 AM. The PWT increased to 8.90 ± 1.07 g at noon and reached a maximum at 16:00 h at 9.87 ± 1.16 g. After that, they decreased to obtain the lowest level of PWT of 4.10 ± 0.52 g at 4:00 h. The rhythmic analysis of the time course of this group presented a period of 22.38 ± 0.94 h, an acrophase at 14.06 ± 0.52 h, an amplitude of 3.03 ± 0.25 g, and a mesor of 5.98 ± 0.25 g (Figure 1C). The mean adjusted Sx.y values from each rat were 2.79 ± 0.37 and R^2^ 0.81 ± 0.042. The LD (Figure 1A) and DD groups (Figure 1C) presented rhythmic behavior. Still, the analysis of the measured rhythmic parameters between the groups revealed that the DD group had a reduction in the amplitude (amplitude LD = 4.81 ± 0.48 g vs. DD = 3.03 ± 0.25 g, mean difference = 1.78, *p* = 0.0289, n = 12 vs. n = 8, ANOVA followed by Fisher test), and in the mesor (mesor LD = 10.49 ± 0.45 g vs. DD = 5.989 ± 0.25 g, mean difference = 4.5, *p* < 0.0001, n = 12 vs. n = 8, ANOVA followed by Fisher test) compared to the LD group of rats (Figure 1G,H). In contrast, the DD group’s acrophase and period were not different from those of the LD group (Figure 1F,I). 

Next, a small group of animals (n = 4, Figure 1D) from the DD group returned to the normal LD cycle for 30 days and was tested for the PWT (Figure 1D LDR). The initial value was 4.21 ± 0.03, and the maximum value of 14.52 ± 2.16 values were very similar to the LD control group (5.81 ± 0.38 and 15.49 ± 1.46, respectively). The rhythmic analysis of the time course of this group presented a period of 24.5 ±2.06 h and acrophase at 13.46 ± 0.09 h, an amplitude of 4.62 ± 0.33 g, and a mesor of 8.62 ± 0.58 g. The mean adjusted Sx.y values from each rat were 2.52 ± 0.76 and R^2^ 0.84 ± 0.063. The rhythmic parameters were like those of the control LD group, indicating recovery via the shift in the LD cycle (Figure 1F–I).

Finally, we challenged the rhythmicity of the PWT by shifting the clock time by two hours (LDS, Figure 1E). The light started at 10 h and the experiment at 8 AM, and it finished at the same time the next day. This group (n = 10 rats) had a PWT of 6.96 ± 0.69 g at the beginning of the experiment at 8 AM, which increased to 16.40 ± 1.73 g 16 h later, and decreased to reach the level of 9.69 ± 0.64 g at 4 h the morning. Further rhythm analysis of the time course of the PWT revealed a period of 23.78 ± 0.35 h, an acrophase at 16.55 ± 0.46 h, an amplitude of 4.39 ± 0.76 g, and a mesor of 10.9 ± 0.74 g. The mean adjusted Sx.y values from each rat were 2.24 ± 0.39 and R^2^ 0.82 ± 0.041. The adjusted period value was not statistically different for a 24 h theoretical mean (t = 1.617, df = 9, *p* = 0.5523, one-sample *t*-test). Additionally, as it can be seen in Figure 1F–I, from the four values from the rhythmic parameters from the rhythm analysis, only acrophase in the LDS group was statistically different from that of the control LD group (acrophase LD = 14.63 ± 0.33 vs. LDS = 16.55 ± 0.46, mean difference = −1.926, *p* = 0.0014, n = 12, ANOVA followed by Fisher’s test) indicating that the shifted group retained its rhythmic behavior despite the experimental maneuver. 

### 3.2. Circadian Rhythmicity of Lumbar DA and DOPAC Content and Turnover

To understand the role of DA in maintaining the rhythmicity of PWT in the spinal cord, first, we analyzed if the DA and DOPAC contents and DA turnover in the spinal lumbar have fluctuations throughout the day. As shown in Figure 2A, the lumbar DA content had a daily peak of 52.87 ± 7.24 pg/mg protein at 18:00 h, increasing from 13.95 ± 1.53 pg/mg protein at 6:00 h. The rhythmic analysis of the time course of this group presented an amplitude of 16 ± 1.79 g, a period of 22.54 ± 2.04 h, a mesor of 27.92 ± 0.95, and an acrophase at 17 ± 0.55 h. The mean adjusted Sx.y values from each rat were 5.35 ± 1.10 and R^2^ 0.78 ± 0.10. The period value was not statistically different for the theoretical value of 24 (t = 0.7129, df = 3, *p* = 0.5273, ns, n = 4, one-sample *t*-test). In the same way, but with different time courses, the main DA metabolite DOPAC also had daily changes (Figure 2B). The main peak was 22.92 ± 3.96 pg/mg protein at 10:00 h, increasing from 1.75 ± 0.17 pg/mg protein at 6:00 AM (Figure 2B). The rhythmic analysis of the time course of this group presented an amplitude of 8.93 ± 1.09 g, a period of 23.50 ± 2.36 h, a mesor of 9.11 ± 1.33, and an acrophase at 12.31 ± 0.34 h. The mean adjusted Sx.y values from each rat were 6.69 ± 1.60 and R^2^ 0.77 ± 0.085. The period value was not statistically different to the theoretical value of 24 (t = 0.2116, df = 3, *p* = 0.846, ns, n = 4, one-sample *t*-test). The time course of DA turnover, determined as the relationship between metabolite and neurotransmitter, had a similar feature following the DOPAC course. The main peak was 0.741 ± 0.126 at 10:00 h, increasing from 0.128 ± 0.014 at 6:00 AM (Figure 2C). The rhythmic analysis of the time course of this group presented an amplitude of 0.27 ± 0.038, a period of 22.75 ± 1.70 h, a mesor of 0.211 ± 0.30, and an acrophase at 11.43 ± 0.20 h. The mean adjusted Sx.y values from each rat were 0.23 ± 0.07 and R^2^ 0.71 ± 0.31. The period value was not statistically different for the theoretical value of 24 (t = 0.7346, df = 3, *p* = 0.515, ns, n = 4, one-sample *t*-test). The DA and DOPAC spinal cord content and turnover had rhythmicity in the lumbar spinal cord (Figures in panel D). Interestingly, the acrophase from the PWT of the LD control group, at 14.56 ± 0.40 h (Figure 1A,I), was closer to the DOPAC acrophase (12.31 ± 0.34) and turnover acrophase (11.43 ± 0.20) than that of the DA acrophase (17 ± 0.55).

### 3.3. Effect of 6-OHDA Lesion of A11 on Paw Withdrawal Threshold

The dopaminergic A11 area has been indicated as the main source of DA in the spinal cord [11,12,13,14,15]. In this study, we analyzed if the 6-OHDA lesion of A11 modifies lumbar DA content and if it changes PWT circadian rhythmicity. First, we evaluated the loss of TH dopaminergic neurons of the A11 nucleus eight days after the injection of 6-OHDA. In Figure 3A, a schematic drawing of the location of the A11 area is shown, followed by two microphotographs of TH staining in this area—one on the control side and the other on the A11 area lesioned side. The reduction in the TH-positive elements as a percentage of control was 48 ± 5% (t = 9.09, df = 4, *p* = 0.008, n = 3 lesioned rats) compared with the control group (Figure 3B) correlated with the loss of DA content in the lumbar region. We measured the effect of lesions in separated animals at 8:00 and 18:00 h (Figure 3C) to evaluate changes in DA concentration during the low and maximum levels according to the rhythmic data (Figure 2A). During the day, the DA content decreased by 42 ± 6% compared to the control values (t = 6.746, df = 8, *p* = 0.0001, n = 5 animals), and in the same fashion, during the night, the DA content decreased by 46 ± 9% with respect to the control (t = 5.09, df = 8, *p* = 0.275, n = 5 animals). 

The impact of the A11 area lesion with 6-OHDA on PWT was analyzed. We used two groups of animals to evaluate the PWT: one group of rats with an A11 lesion bilaterally injected with 6-OHDA (10 nM/µL) and the other injected with the vehicle solution (Figure 3D). Both groups were maintained in LD cycles (12 h–12 h). The evaluation of the PWT was carried out every 4 h for 24 h in all groups. Rats in the intrahypothalamic saline-injected group (Figure 3D, n = 10 rats) had PWT values of about 8.42 ± 3.18 g at the beginning of the experiment (8:00 h). This value increased to about 17.68 ± 3.18 g at 12 h (4 h later), and after that, it decreased to 4.07 ± 0.03 g at 4:00 h in the morning. Further rhythm analysis of the time course of the PWT revealed a period of 23.50 ± 0.80 h, an acrophase at 13.97 ± 0.41 h, an amplitude of 5.82 ± 0.50 g, and a mesor of 9.72 ± 0.72 g. The mean adjusted Sx.y values from each rat were 4.06 ± 0.95 and R^2^ 0.82 ± 0.05. The rhythmicity values from the saline group did not differ statistically when compared with the LD control group (see Figure 1D, LD period = 23.78 vs. vehicle = 23.50 ± 0.80, t = 0.352, df = 14, *p* = 0.727; LD acrophase = 14.56 ± 0.40 vs. vehicle = 13.97 ± 0.41, t = 0.0.966, df = 14, *p* = 0.3501; amplitude LD group = 5.04 ± 0.55 vs. vehicle = 5.82 ± 0.50, t = 0.925, df = 14, *p* = 0.357; mesor LD group = 10.95 ± 0.40 vs. vehicle = 9.72 ± 0.72, t = 1.59, df = 14, *p* = 0.1322, unpaired Student’s *t*-test). The mean period value obtained was not statistically different for the 24 h theoretical mean (t = 0.61, df = 5, *p* = 0.566, one-sample *t*-test). In contrast, the intrahypothalamic A11 nucleus injection of 6-OHDA produced a severe decrement in the PWT threshold after eight days of the 6-OHDA injection at 12:00 and 4:00 PM compared with the control vehicle group at 8, 12, 16, and 20 h (Figure 3D, red points vs. black points and line). Moreover, the intrahypothalamic injection of 6-OHDA abolished the circadian rhythm of the PWT since all PWT values remained low along the clock time cycle (Figure 2D red points), and the data could not be adjusted to a sinewave with enough goodness. The mean adjusted Sx.y values from each rat were 13.91 ± 5.33 and R2 0.48 ± 0.13. 

### 3.4. Intrathecal DA Receptor Antagonist Changes PWT Rhythmicity

To assess the role of spinal DR in the modulation of the circadian PWT in naïve rats, we studied the effect of the intrathecal injection of D1-like (SCH-23390, 3.24 µg) and D2 (L-741,626 3.4 µg) antagonists in two groups of rats, while control groups received the vehicle solution for drugs. The animals received a daily injection of each drug for three consecutive days; on the fourth day, the time course of the PWT was evaluated every 4 h for 24 h as previously described. In the control group, the vehicle for drugs was administered (see upper panel Figure 4). The vehicle group had a PWT value of about 7.55 ± 0.65 g at the beginning of the experiment (8:00 h). This value increased to about 18.80 ± 1.16 g at noon (4 h later), and after that, it decreased to 7.74 ± 1.01 g at the end of the experiment (4:00 h in the morning). The rhythmicity of this control group was similar to the DL control group (Figure 1A) since their adjusted parameters to the sinewave curve were not statistically different (DL group period = 23.78 ± 0.35 vs. vehicle intrathecal group = 22.18 ± 0.87, t = 1.728, df = 18, *p* = 0.10; DL amplitude = 5.04 ± 0.55 vs. vehicle intrathecal group = 5.56 ± 0.31, t = 0.8069, df = 18 *p* = 0.403; DL group mesor = 10.95 ± 0.40 vs. vehicle intrathecal group = 10.85 ± 0.75, t = 0.1105, df = 18, *p* = 0.91; DL group acrophase = 14.56 ± 0.40 vs. 13.28 ± 0.73, t = 1.52, df = 18, *p* = 0.149). The mean adjusted Sx.y values from each rat were 5.16 ± 0.72 and R^2^ 0.83 ± 0.03.

The effect of the D1-like antagonist SCH-23390 (10 nM/5 μL) was studied in five animals and compared with the intrathecal vehicle group (Figure 4A). The drug administration significantly decreased the PWT values during the light phase at 8, 12, and 16 h (values). An unexpected finding was the large change in rhythmicity produced by the antagonist since all sinewave parameters were modified. The amplitude decreased in the control from 5.56 ± 0.31 g to 2.24 ± 1.10 g (mean difference = 4.26, *p* < 0.001, Figure 4D), in the mesor from 10.85 ± 0.75 to 5.07 ± 0.62 g (mean difference = 5.77, *p* < 0.001, Figure 4F), in the acrophase from 13.28 ± 0.73 to 6.90 ± 1.19 h (mean difference = 6.37, *p* < 0.001, Figure 4E), and in the period from 21.8 ± 0.68 to 14.52 ± 1.07 h (mean difference = 5.95, *p* < 0.001, n = 10, ANOVA followed by Tukey, Figure 4C). This draws attention to the fact that that the fall in the period is almost half the control group (Figure 4C). However, unlike for the A11 lesion (Figure 3D), sinewave adjustment was possible (the mean adjusted Sx.y values from each rat were 1.62 ± 0.43 and R2 0.79 ± 0.06). On the other hand, the L-741,626 group (D2 receptor antagonist (10 nM/5 µL)) had decreased PWT values at most of the hours measured except at 20 h (Figure 4B). Unlike the D1 antagonist, the D2 antagonist only modified significant values related to the PWT, such as amplitude and mesor, but retained rhythmicity. The amplitude decreased in the control from 5.56 ± 0.31 to 2.59 ± 0.53 g (mean difference = 2.97, *p* < 0.001, Figure 4D) and in the mesor from 10.85 ± 0.75 to 5.30 ± 0.61 g (mean difference = 5.54, *p* < 0.001, Figure 4F).

### 3.5. A11 Lesions and D1-like and D2 Receptor Antagonist Change Spinal Cord Clock Gene Expression

We evaluated the effect of the A11 lesion and D1 and D2 intrathecal receptor antagonists to investigate if DA regulates clock gene expression as changes are induced in the PWT rhythms. Eight days after the 6-OHDA injection, and after 4 days of D1-like and D2/D3 receptor blockade, the rats were sacrificed, their lumbar spinal cords extracted, and the expression of several spinal clock genes determined. The results indicated that the 6-OHDA A11 lesion induced a decrement in Clock and Per1 gene expression and an increment in Per2 expression during the day (Clock: 0.72 ± 0.16, t = 3.447, *p* = 0.041, Per1: 0.52 ± 0.05, t = 0.614, *p* = 0.0087; Per2: 2.25 ± 0.195, t = 6.421, *p* = 0.0077, n = 4 animals, one-sample *t*-test); the rest of the clock genes evaluated did not show significant differences. Contrariwise, the lesion induced a decrement in Clock, Bmal, Per1, Per2, Per3, Cry1, and Cry2 in the night (Clock: 0.47 ± 0.11, t = 4.73, *p* = 0.0179; Bmal: 0.37 ± 0.07, t = 8.33, *p* = 0.0036; Per1: 0.525 ± 0.047, t = 9.92, *p* = 0.0022; Per2: 0.30 ± 0.040, t = 17.15, *p* = 0.004; Per3: 0.57 ± 0.047, t = 4.49, *p* = 0.0206; Cry1: 0.35 ± 0.064, t = 10.07, *p* = 0.0021; Cry2: 0.40 ± 0.08, t = 7.348, *p* = 0.0052, n = 4, one-sample *t*-test, Figure 5A). 

The D1-like receptor blockade induced variable changes in the expression of clock genes, as seen in Figure 5B. Clock and Per2 decreased during the day and night, and Cry2 only during the day (Clock during the day: 0.466 ± 0.088, t = 6.047, *p* = 0.0263; Clock at night: 0.2667 ± 0.55 t = 0.11, *p* = 0.0082; Per2 during the day: 0.50 ± 0.13, t = 3.77, *p* = 0.063; Per2 at night: 0.55 ± 0.07, t = 5.71, *p* = 0.029; Cry2 during the day: 0.5 ± 0.13, t = 8.66, *p* = 0.013). Additionally, during the day and night, increments in the expression were observed in Bmal (day: 1.30 ± 0.057, t = 5.196, *p* = 0.0351; night: 1.50 ± 0.09, t = 5.77, *p* = 0.028) and Per1 (day: 1.29 ± 0.006, t = 44, *p* = 0.0005; night: 1.38 ± 0.03, t = 11, *p* = 0.008). Finally, a decrement in Cry1 was only observed at night (0.50 ± 0.1, t = 5.00, *p* = 0.0377). The D2 receptor antagonist increased the expression of Bmal, Per2, and Per3 during the day and night (Figure 5C). The increment in the expression of Bmal at night was much higher than during the day (during the day: 2.0 ± 0.20, t = 5.00, *p* = 0.0377; at night: 4.56 ± 0.63, t = 5.608, *p* = 0.030 with respect to one, n = 3, one-sample *t*-test). On the other hand, increments in Per2 and Per3 were very similar for both determinations (Per2, day: 2 ± 0.115, t = 8.66, *p* = 0.0131 and night: 2.33 ± 0.29 t = 4.50, *p* = 0.0460 with respect to one; Per3, day: 2.033 ± 0.20, t = 5.096, *p* = 0.0364 and night: 2.367 ± 0.272, t = 5.09, *p* = 0.0376, n = 3 vs. one; one-sample *t*-test). Decrements were only found in Per1 for both determinations during the day (0.48 ± 0.10, t = 4.42, *p* = 0.05) and night (0.50 ± 0.056, t = 5.06, *p* = 0.03).

## 4. Discussion

This study reveals the physiological rhythmic properties of the PWT of the spinal cord. Additionally, we demonstrate that the dopaminergic A11 nuclei modulate the PWT rhythm through the activity of the spinal D1-like and D2 receptors, and that A11 and spinal DR modulate clock gene transcription in the spinal cord. 

### 4.1. Rhythmicity of PWT in Normal Rats

Our results showed a circadian rhythm in the PWT of male Wistar naïve rats (Figure 1A). The daily changes in PWT had the characteristics of a circadian rhythm: a near-24 h period with defined acrophase, amplitude, and mesor values. This was an authentic rhythm since it was constant over time, as shown by the determination of very similar values of the period, mesor amplitude, and acrophase one month later (Figure 1B,F–I). Some notable features of circadian rhythm apply to PWT rhythmicity [38]. First, it persisted even in the continuous darkness condition (Figure 1C) since no changes in the period or acrophase occurred (Figure 1F,I); however, the amplitude and mesor decreased (Figure 1G,H). These decrements indicate that the PWT values are also determined by light exposure and controlled by the suprachiasmatic nucleus (SCN) [39]. Second, although continuous darkness did not modify the acrophase, shifting light onset increased it, indicating a phase dependence of the circadian rhythm (Figure 1I). Third, after the reinstallation of light (after the continuous darkness exposure period), the rhythmic parameters (period and acrophase) remained near the control values (Figure 4F,I). Significantly, the amplitude and mesor recovered, reinforcing that light determines the threshold values. Changes observed during the four experimental conditions of the PWT daily changes strengthen the assumption that the PWT threshold has a circadian rhythm. Additionally, these findings indicate that circadian PWT behavior is inherent in the spinal cord since rats evaluated a month after that had the same rhythmic parameters. In addition, it could be proposed that the circadian rhythm of the PWT is subjected to the SCN’s influence since light shifting modifies SCN activity [40] and changes the acrophase value, and the reinstallation of light after a DD period maintains rhythmicity. This is a logical result because the organization of the biological systems in mammalian rhythms is mainly controlled by the SCN [39], and the peripheral clock genes outside the SCN have intrinsic activity; thus, they can independently maintain circadian activities [1,9,41].

Rodents’ motor activity has a circadian rhythm, with higher activity in the dark phase and minor in the light [2]. We found that rats are more sensitive to mechanical stimulation (lower PWT values) when they are more active than during the day when they are less active. This can sound paradoxical, but it could be expected if it is assumed that there is a positive relationship between activity and high values of PWT. This is expected since a correlation between activity and high values of PWT seems feasible. Hypersensitivity during the night phase or around it has been observed in other modalities of perceptions, particularly those related to pain; for example, thermal stimulation in the hot-plate test elicits more sensible responses during the last hours of the night and first of the day (4:30–11:30 h) [3]. Our experimental data indicate that higher values of PWT occur between 4:00 and 8:00, probably until 10:00 h. Likewise, others have reported more sensitivity to thermal or chemical (1% formalin) stimuli in mice during the night [4,42,43]. Interestingly, in the tail-flick test, pain sensitivity in C57BL/6 mice is higher at night, contrasting with Swiss mice, which are the opposite [4]. In another report, ICR mice (Charles River Japan) had no evident mechanonociception rhythmicity measured in control conditions with von Frey filaments; however, after sciatic nerve ligature, PWT rhythmicity became evident with higher values from dawn to noon (16 to 21 h) [44,45]. Although some of this evidence came from mouse species, it reinforces the idea that genetic factors and environmental and pathophysiological conditions are determinants of mechanonociception sensitivity and rhythmicity. 

Our results show that PWT values vary between 5 and 12 g. Interestingly, despite having healthy rats, the PWT threshold reached values near those considered in a neuropathic condition < 4 g [25]. Central and peripheral neuropathic pain has allodynia and hyperalgesia as its hallmarks [46]. Hyperalgesia is an exacerbated response to increased pain from a nociceptive stimulus, and allodynia is defined as the pain perception of a nonpainful stimulus [47]. It has been demonstrated that rat and mouse neuropathic pain models have rhythms with increased sensitivity during the day [45,48], unlike what was observed in our experimental condition. Thus, we conclude that the rats used in this study have mechanical hypersensitivity without allodynia with a circadian rhythm, but showed no neuropathic pain condition. We conclude this because: first, our rats had lower values of PWT in response to a mechanical stimulus but not at levels considered neuropathic, at least in the normal condition [25]; second, our rats did not have a neuropathic pain condition since they are healthy; and third, the PWT rhythmicity determined in our rats is contrary to the PWT rhythmicity shown in neuropathic pain rodents [45].

### 4.2. Dopaminergic Dependence of PWT Rhythms and Their Threshold

It has been shown that the content of DA in different brain structures (retina, olfactory bulb, midbrain, striatum, and hypothalamus, among others) has circadian rhythmicity. Herein, DA content exhibits a peak during the day [49,50,51,52,53,54]; in contrast, changes in extracellular DA concentration measured using microdialysis, particularly in the striatum, occur at night [55,56,57,58,59,60]. Such differences between the content and extracellular content could result from the activity of transporters [58] or DA utilization [61]. Since we cannot technically measure extracellular DA using microdialysis in the lumbar region, we measured DA and its principal metabolite DOPAC to obtain an idea of a possible correlation of the daily changes in DA and DOPAC contents with the changes in PWT in the lumbar region (Figure 2). In our experimental conditions, as reported, DA content had a circadian rhythm, with an acrophase at 17:00 h. This peak partially agrees with DA content reports in other SNC areas and our previous report on the spinal cord [24], but does not correlate with the rhythms of the PWT since acrophase is significantly higher (Figure 2D). Thus, it seems that DA and DOPAC contents had a circadian rhythm that was out of phase concerning the PWT rhythm. Nevertheless, DA turnover, evaluated as the ratio of DOPAC/DA, had values of the acrophase, which were not different from the PWT acrophase (Figure 2D). These turnover values have similar rhythmicity to those determinations reported upon inhibition of TH in the Wistar rat brain, whose peaks occur between 12:30 and 16:30 h during the day [61] and DOPAC/DA and whose ratios have higher values during the day in the olfactory bulb [51]. Unfortunately, we cannot properly evaluate the correlation of turnover and PWT rhythmic data by statistical means since the determination of DA and DOPAC contents was measured at different times. However, we think that the correspondence of the rhythmicity of both parameters strongly suggests that the high PWT values correlate with a higher DA turnover. The high turnover values near 12 h (Figure 2C), in correspondence with the higher PWT values (Figure 1A), indicate a lack of acrophase differences (Figure 2D) and are in line with the antinociceptive effect of DA [16,19]. There is strong evidence of a descending dopaminergic pathway to the lumbar spinal cord from the A11 area [11,12,13,14,15]. Thus, as expected, selective 6-OHDA injections in the A11 area decrease TH-positive elements (Figure 3A,B [62]) and significantly reduce the DA content in the lumbar spinal cord (Figure 3C [63]). 

Two main functions have been attributed to the A11 area’s descending innervation to the spinal cord. One is related to motor locomotion, and the other to pain perception. DA contributes to locomotor activity modulating the activity of CPG networks [64]. DA and D1-like receptor activation is enough to promote locomotor activity in rats [65]. Additionally, D2 receptors can decrease the efficacy of recurrent collateral feedback from motoneurons affecting CPG circuits. DA can potentiate network activity while simultaneously reducing the gain of recurrent excitatory feedback loops from motoneurons onto the network [66]. In fact, the optogenetic stimulation of A11 vGAT and vGluT2 dopaminergic neurons initiates and modulates locomotion. DA, through D1/D5 receptors, enhance stability, whereas D2, D3, and D4 slow the rhythm [67,68]. On this basis, one could expect that the A11 lesion decreases locomotor activity; however, the 6-OHDA lesion induced an increment in locomotor activity, particularly in the traveled distance [63]. Thus, changes in PWT values cannot be related to immobility or, most probably, to the activity of DR differentially distributed across the transverse spinal cord. D2-like receptors are distributed mainly in lamina I-III of the dorsal horn, where they mediate antinociceptive effects, and D1-like receptors are most strongly expressed in the ventral horn where motor circuits reside [17]. However, the D2-mediated inhibition of primary afferents and postsynaptic neurons in the substantia gelatinosa indicates that the anti-nociceptive effect occurs at the dorsal horn, which could activate motor circuits to promote motor function [69].

An interesting finding in our research is the notable effect exerted by the A11 lesion on PWT rhythmicity and values, as shown in Figure 3D, all day long. PWT values move to tactile allodynia, and no rhythmic parameters can be adjusted. These findings indicate the dopaminergic regulation of PWT circadian rhythm and, as expected, its value in the A11 area. The principle of PWT rhythmicity and the modulation of its sensitivity by the dopaminergic neurons of the A11 area are undoubtedly related to its function as a sensory system that selectively responds to mechanosensitive and visual stimulation [70], whose projections to the spinal cord are modulated by DR mechanoreceptive afferents [21]. Recent data indicate a differential role of dorsal horn neurons in the generation of allodynia. Calretinin (CR) neurons in the lamina II inner convey mechanical allodynia induced by inflammatory injuries, while protein kinase C gamma (PKCγ) neurons at the lamina II/III border convey mechanical allodynia induced by neuropathic injuries. Cholecystokinin (CCK) neurons located deeper within the dorsal horn (laminae III-IV) are key for both types of injury [71]. Although the A11 area lesion is not a proper spinal cord injury, the lack of DA possibly determines the modification of one of these circuits. If we consider that DA denervation can be taken as a spinal injury due to the decrement of dopaminergic axons onto mechanonociceptive neurons, then the PKCγ neurons are an excellent candidate to study the action of DA in these neurons.

The literature data also indicate that tonic dopaminergic activity has an antinociceptive role in the lumbar spinal cord and in other areas [16] or other pain modalities such as trigeminal analgesia [72] and migraine [14] related to the A11 area. Interestingly, a 6-OHDA lesion of the A11 area has been proposed as a Restless Legs Syndrome (RLS) model [62], an entity with circadian rhythms in its symptomatology [73] and in which there is a prominent role of DA [74,75]. Patients with this disease suffer an unpleasant component attributed to dysfunction of the intrinsic antinociceptive mechanism [76]. In addition, RLS patients show mechanical hyperalgesia but not allodynia [76]; thus, our data could suggest that tactile hyperalgesia after the A11 lesion could result from the dysfunction of DA action on the projection areas of A11 dopaminergic neurons. In summary, dopaminergic A11-area neurons projecting to the spinal cord modulate the threshold and rhythmicity of mechanonociception. 

### 4.3. Role of D1-like and D2 Receptors on PWT Values and Its Rhythmicity

In this study, we analyzed the effect of the lumbar blockade of D1-like and D2 receptors to determine if the A11 lesion effect is due to a loss of function in the DR. Although both receptors decreased PWT values near allodynia, they turned openly allodynic at some time points of the day (Figure 4). Still, the effect was different concerning the PWT circadian rhythm. D2 blockade decreased the values of amplitude and mesor but retained the period and acrophase values. In contrast, the D1-like receptor blockade had a lower amplitude, mesor, period, and acrophase than previous normal conditions. 

Significant evidence supports the expression, particularly in the dorsal horn of the spinal cord, of the D1-like and D2 receptors [17]. The low PWT values observed after the intrathecal administration of SCH-23390, a D1-like antagonist, suggest the modulation of PWT at two levels—one at the PWT value and the other at the circadian rhythm level. According to our data, D1-like receptors seem to have an anti-mechanical hypersensitivity or anti-hyperalgesic role in this typical condition. Some electrophysiological and behavioral evidence favors a pro-hypersensitivity and -hyperalgesia role of D1 receptors in the spinal cord. D1-like receptors produce LTP on C fibers through cAMP, which could contribute to hyperalgesia via an intense noxious stimulatory stimulus [77]. In line with the hyperalgesic role, lumbar D5 receptors induced mechanical hypersensitivity [78,79]. However, it should be considered that this effect occurs in models in which pain priming was previously induced, suggesting a role in pain development and maintenance. On the other hand, DA via D1 receptors depresses slow ventral root potential [80] by acting on interneurons; accordingly, it could be expected to have an analgesic effect. Thus, the action of D1-like receptors in pain seems complex and probably happens in the same way with the modulation of mechanonociception. More research is necessary to clarify their role in both entities properly. 

Several studies indicate an analgesic role of D2 receptors in the spinal cord, particularly in mechanonociception [81]. For example, the D2 receptor agonist, iontophoretically applied, prevents an inhibitory response to nociceptive stimuli in spinothalamic, spino-mesencephalic, and spino-cervical neurons [19], and also inhibits substantia gelatinosa neurons [69]. Moreover, the D2-like receptor agonist quinpirole intrathecally increases PWT in normal rats, indicating an antimechanonociceptive effect in normal rats [82]. Thus, contrary to the D1-like receptor, the D2 receptor has a modulatory anti-hypersensitive mechanonociception role in line with antinociceptive action. As L 745,626 mainly has an effect on D2 and D3 receptors that have been involved in pain perception, the relative contribution to the rhythmicity and threshold of mechanonociception of subtypes of the D2-like receptor should be explored. Recent evidence indicates that D4 receptors in primary Aɗ and C fibers decrease EPSC inhibition of the proprioceptive signal [83]; thus, D4R also needs to be explored. Interestingly, the D2-like decrement in high-voltage Ca^2+^ currents in DGR neurons is mediated by D4 receptors [84]. 

Interestingly, a recent study showed that in a neuropathic pain model, the blockade of D1 and D2 receptors (separately) inhibits the D1–D2R dimer complex, producing mechanical hypersensitivity [85]. Despite the implication of neuropathic pain, this fact suggests a synergistic role of D1 and D2 receptors in altered mechanonociception during pain. Thus, such synergistic action may occur in normal conditions. Additionally, in the pineal gland, variations in D4 and adrenergic receptor heteromerization modulate circadian variations in melatonin synthesis and release [86]. Thus, it is possible that circadian variations in D1–D2 receptor dimerization modulate PWT rhythms. The A11 6-OHDA lesion seems to reflect changes in PWT values via the lack of activity of D1 and D2 receptors.

Our results show that the lesion of dopaminergic A11 neurons modifies the transcription profile of several spinal clock genes, including the primary genes that regulate circadian rhythms—*Clock*, *Bmal*, *Per1*, *Per2*, *Per3*, *Cry1*, and *Cry2*—especially during the night phase when PWT values are lower (Figure 1A). These results suggest that the integrity of the A11 neurons is not only needed to preserve the expression of the spinal clock genes, but also, in turn, to maintain the circadian rhythm parameters of mechanonociception. The intracerebroventricular injection of 6-OHDA in rats disrupts *Per2* expression (blunted) in the rat forebrain [87]. Similarly, in intrastriatal 6-OHDA-treated rats and an MPTP mouse model lesioned in the substantia nigra, altered expression of the circadian rhythms of *Clock*, *Bmal*, *Per*, and *Cry* were reported in the SCN and striatum [88,89]. These data indicate that in several brain areas, the loss of DA action modifies the expression and rhythms of clock genes; thus, this is the same with respect to the spinal cord and its dopaminergic innervation from the A11 area. These effects of DA on clock gene expression can be related to altered mechanonociception in RLS [75]. Similarly, clock gene alteration in Parkinson’s disease, produced by the loss of DA, might contribute to the development in the symptomatology of the condition via altered circadian rhythms [90]. 

In the SCN [91], D1 receptor activation affects clock genes differently. It increases *Per2* and *Clock* and decreases *Per1* and *Bmal* during the day; at night, it increases *Per2* and reduces *Per1*, *Clock*, and *Bmal*. It is expected that the blockade of D1 receptors produces opposite effects. In the lumbar region, we found that the D1-like antagonist, during the day, decreases *Per2*, *Clock*, and *Cry2* and increases *Per1* and *Bmal*, whereas at night, it decreases *Per2*, *Clock*, and *Cry1* and increases *Bmal* and *Per1*. This is almost a mirror image concerning the SCN, except for the *Clock* gene, but it indicates that the D1 receptor modulation of clock gene expression occurs similarly to in the SCN. In the SCN, the changes in these clock genes are related to motor activity [91]; in the lumbar region, the D1 receptor modulation of gene expression in the lumbar region is associated with PWT rhythm and its value. In our data, the altered expression observed in *Clock*, *Bmal*, and *Per1* could be related to the shortness of the period and acrophase since the effect was not observed with the D2 receptor blockade, where no changes in *Clock* were found. There, the rhythmic parameters period and acrophase were maintained. Thus, the D1 receptor, through regulation of this clock gene, determines the period of the rhythmic oscillation of PWT, whereas the regulation of the mechanoreceptive afferent signals the PWT values. 

In the case of D2 receptors, a decrease in *Clock*, *Per1*, and increments in *Per2* in the striatum have been reported [6,7]. On the other hand, D2 receptors increase *Bmal*, *Clock*, and *Per1* expression in the retina [8]; thus, changes in clock genes due to D2 receptors depend on the studied region. Considering that the blockade of D2 receptors should produce the opposite of activation, we can propose that D2 receptors decrease *Bmal*, *Per2*, and *Per3* and increase *Per1* throughout the day. D2 receptor signaling enhances the transcriptional activity of the Clock–Bmal complex via MAPK in the retina, which induces *Per1* transcription by relieving the *Cry1* repression of *Clock* [8]. Thus, in our experimental conditions, the D2 receptor blockade could decrease the transcriptional activity of the complex and the expression of *Per1*. Similarly, in the striatum, endogenous DA via daily activation of D2 receptors regulates *Per2* expression at night [7], indicating that D2 receptors modulate clock Per gene expression. Morioka et al. [1] showed that the downregulation of the *Per1* gene in the spinal dorsal horn in astrocytes and neurons leads to mechanical hyperalgesia via JNK and CCL2 production. In our study, the D2 receptor blockade produces a decrement in the expression of *Per1*. Thus, mechanical hyperalgesia, shown by the low values of PWT after the D2 blockade, could be caused by the change in *Per1* expression and its effects on JNK signaling. Additionally, the lack of changes in *Clock* gene expression could be related to the maintenance of the period and acrophase. More research is necessary to establish the precise relationship between DR activity and functional changes. Moreover, since changes in clock gene activity modify dopaminergic transmission [10], there is a reciprocal regulation of both systems, which should be considered. Thus, DR modulates PWT rhythmic activity throughout the modulation of clock gene expression. 

## 5. Conclusions

Our study showed a circadian rhythm in the mechanonociception modulated by DA D1 and D2 receptors, activated by dopaminergic afferents from the A11 nuclei. This regulation could be relevant for RLS. 

## Data Availability

The data are available from the corresponding author upon reasonable request.
